# On-Device IoT-Based Predictive Maintenance Analytics Model: Comparing TinyLSTM and TinyModel from Edge Impulse

**DOI:** 10.3390/s22145174

**Published:** 2022-07-11

**Authors:** Irene Niyonambaza Mihigo, Marco Zennaro, Alfred Uwitonze, James Rwigema, Marcelo Rovai

**Affiliations:** 1 African Centre of Excellence in Internet of Things, College of Science and Technology, University of Rwanda, Kigali P.O. Box 4285, Rwanda; 2 Telecommunications/ICT4D Laboratory, The Abdus Salam International Centre for Theoretical Physics, Strada Costiera, 34151 Trieste, Italy; 3 College of Science and Technology, University of Rwanda, Kigali P.O. Box 4285, Rwanda; alfruwitonze@gmail.com (A.U.); jamesrwigema@gmail.com (J.R.); 4 Instituto de Engenharia de Sistemas e Tecnologia da Informação, Universidade Federal de Itajubá, Itajuba 37500-903, Brazil; rovai@unifei.edu.br

**Keywords:** predictive maintenance, edge, maintenance actions, remaining useful life, equipment, TinyModel, real-time data

## Abstract

A precise prediction of the health status of industrial equipment is of significant importance to determine its reliability and lifespan. This prediction provides users information that is useful in determining when to service, repair, or replace the unhealthy equipment’s components. In the last decades, many works have been conducted on data-driven prognostic models to estimate the asset’s remaining useful life. These models require updates on the novel happenings from regular diagnostics, otherwise, failure may happen before the estimated time due to different facts that may oblige rapid maintenance actions, including unexpected replacement. Adding to offline prognostic models, the continuous monitoring and prediction of remaining useful life can prevent failures, increase the useful lifespan through on-time maintenance actions, and reduce the unnecessary preventive maintenance and associated costs. This paper presents the ability of the two real-time tiny predictive analytics models: tiny long short-term memory (TinyLSTM) and sequential dense neural network (DNN). The model (TinyModel) from Edge Impulse is used to predict the remaining useful life of the equipment by considering the status of its different components. The equipment degradation insights were assessed through the real-time data gathered from operating equipment. To label our dataset, fuzzy logic based on the maintainer’s expertise is used to generate maintenance priorities, which are later used to compute the actual remaining useful life. The predictive analytic models were developed and performed well, with an evaluation loss of 0.01 and 0.11, respectively, for the LSTM and model from Edge Impulse. Both models were converted into TinyModels for on-device deployment. Unseen data were used to simulate the deployment of both TinyModels. Conferring to the evaluation and deployment results, both TinyLSTM and TinyModel from Edge Impulse are powerful in real-time predictive maintenance, but the model from Edge Impulse is much easier in terms of development, conversion to Tiny version, and deployment.

## 1. Introduction

In today’s maintenance, the early fault detection and prediction is mainly centered on the maintainer’s experience and their familiarity with the equipment. Due to the development in engineering and the complexity of equipment, human judgment is not sufficient to prematurely predict and continuously oversee the status of assets.

As result, over recent decades, machine learning models have been highlighted as meaningful tools in predictive maintenance, especially in prognostic and asset health management [1]. Different prognostic algorithms and models [2] have been developed using simulated [3] and/or offline data sets [4,5,6,7] to estimate the asset’s remaining useful life (RUL), which is based on the targeted values function.

There is no universal model due to different systems, type and quality of data, different performance conditions, and working principles; therefore, the construction of a life degradation model up to total failure is mainly performed for the projection of maintenance actions, especially to keep spare inventory on track ahead of time, and it is based on subjective choice [3], such as asset state, age, usage load, deterioration curve, rate or patterns, failure types, and conducted maintenance history parameters.

In addition, most of the RUL models for traditional regression problems do not operate in real-time; thus, they must be updated by new findings from the regular preventive maintenance [8]. They also mainly rely on the logistical, managerial, and decision support processes to procure the spares regarding the estimated lifetime, rather than the continuous fault diagnostics that may fortify the powerful prediction.

Among others, traditional models, such as random forest and XGBoost [9], Seasonal Autoregressive Integrated Moving Average (SARIMA) [10], Support Vector Machine (SVM) [11,12,13], a stochastic model, such as Hidden Markov Model (HMM) [14], and fuzzy neural network [15] have demonstrated efficient predictions on real-time data, but with some limitations based on manual data processing and expertise of the developer [5,16]. Deep learning has shown admirable performance in the prognosis of a high amount of data [16]. Among the popular deep prognostic models are Convolutional Neural Network (CNN) [5,6,7,11] and Recurrent neural network—particularly its long short-term memory [4,17,18,19,20] version.

In the predictive maintenance domain, the equipment degradation recalls the long-term chronological beliefs from its real-time physical performance data; thus, the long short-term memory (LSTM) variant has been appreciated and adopted in the literature to provide better performance for time series data [9,19,20] due to its capability to preserve information for long periods.

These asynchronous predictive models do not provide a full availability of the equipment’s health state, thus, prompt downtime may occur between two consecutive prediction periods. Consequently, maintainers claim to continuously oversee their asset’s operating bulletin that may fall in different health states before reaching the failure point. There is also a claim on a continuous and autonomous prediction of the RUL of their equipment.

To overcome this deficiency, through the steady novelties in technology, such as the Internet of Things (IoT), the real-time state of the equipment can be observed continuously on edge by employing different sensors to acquire different health information and the analytics predictive model in real-time on edge devices. The IoT-based predictive maintenance [21] may enable users to obtain autonomous real-time updates of their equipment health conditions for timely accurate decision making on maintenance actions.

Adding to the real-time LSTM on edge (TinyLSTM), the Edge Impulse [22] is recently being developed and deployed as a leading Artificial Intelligence (AI) platform that simplifies the development and deployment of machine learning models on the Tiny embedded devices (at the edge) with a possibility of ultra-low power consumption (TinyML).

Driven by the claims on extended up-time and lifespan of equipment, to minimize unplanned downtime and unnecessary preventive maintenance as well as to reduce the frequency of component replacement, along with this work, we comparatively assessed the development and deployment of TinyLSTM and TinyModel from Edge Impulse performances on the real-time data collected from different components of operating equipment with a purpose to advise the best on edge predictive maintenance analytics model. Both have been built and evaluated using Keras library [23]. Later, they are transformed into the TinyModel version for their deployment on the IoT device installed on the monitored equipment.

Adding to existing offline sequential predictive maintenance models, the TinyModel proposed in this work provides real-time updates on the RUL of the running equipment and reduces the maintenance time as well as cost through performing only the necessary maintenance activities in a convenient time; therefore, the model will be continuously running, and the device on the edge will also require a close degradation monitoring for all of its parts.

By considering the maintainers’ expertise, the gathered real-time data were preprocessed using the fuzzy expert system to find the diagnostic facts to label the maintenance actions priority, from which the actual remaining useful life could be calculated depending on the conditional status of each component.

The major contributions of this paper are the following:

- For real-time application, the real-time performance data to develop the model were gathered using an IoT device installed on the equipment.
- For multi-conditional parameters that may separately affect the equipment life, the data are labeled using the fuzzy expert system based on the maintainers’ expertise.
- To predict an equipment’s remaining useful life depending on equipment’s component’s status, the IoT-based real-time predictive analytics models—LSTM and Model from Edge Impulse—are developed and compared.
- For the fault prediction and early notification on maintenance priority suggestions, each model is converted to TinyModel, and its deployment onto an IoT device for continuous real-time health monitoring is simulated.

The results show the prediction ability is good with a low mean squared error (MSE) of 0.01 and 0.11 for LSTM and the model from Edge Impulse, respectively. The R2 for LSTM is 77% whereas the accuracy is 99.87% for the model from Edge Impulse. We compared the process from model creation up to deployment simulation and we concluded by proposing the TinyModel from Edge Impulse platform to be a suitable and easy model for real-time edge application.

The remainder of this paper is organized as follows: we introduce the data gathering and processing in Section 2; Section 3 details the functionalities of selected models; Section 4 discusses models’ results; Section 5 concludes the work and suggests future works.

## 2. Data Gathering and Processing

**Figure 1:**
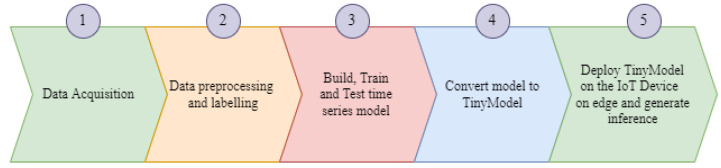
Strategic steps to build an Analytics TinyModel.

Following the defined steps to develop and deploy a predictive TinyModel, as shown in Figure 1, the main Predictive Maintenance (PdM) engine is the data and the way they are preprocessed. The type and quality of data determine the prediction accuracy that reflects the correctness of decisions on maintenance actions to be taken. For better precision in prediction, two types of data are needed: historical and real-time data. Historical data help to understand the operation of the equipment and to select the critical physical condition parameters to be monitored in a real-time manner. They are recorded by the experts in maintenance who also help to determine the necessary parameters to monitor. The real-time data from the specified equipment shall provide clear insights of its real health status. This also requires vigorously observing any change from the real-time data against the behaviors of the equipment operating status.

### 2.1. Data Acquisition

The process to collect the real-time data starts by choosing the equipment, understanding its operational procedures, learning its diagnostics from the maintenance history, and highlighting the major detected faults, their causes, and their impact on the overall equipment life. From the acquired evidence, we highlight the critical components, their conditional parameters to be intelligently observed, as well as the required materials.

**Figure 2:**
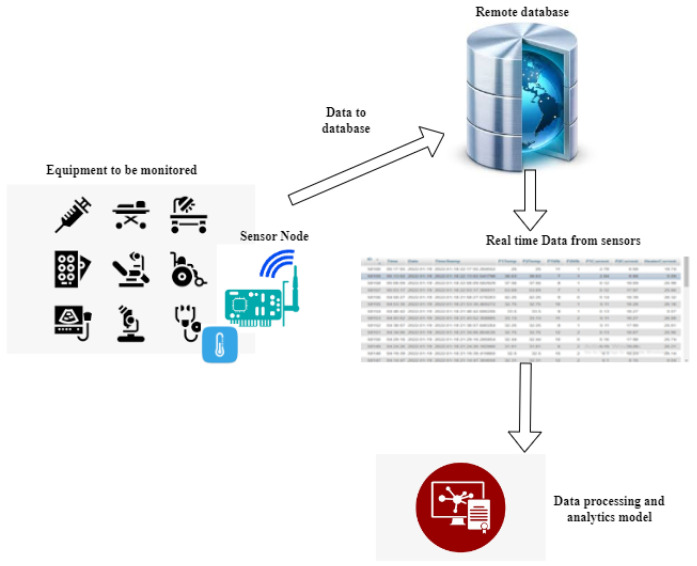
Data acquisition process.

In line with the PdM claim, the longtime real-time data that may provide different scenarios of the equipment’s health status were collected using the data collector developed in [24]. The data collection processes shown in Figure 2 are used in this work to collect and save data before being processed and used to build the predictive model.

The defined sensors according to the critical health condition parameters were installed on different critical components of the selected equipment (autoclave sterilizer) and timestamped data were sent to the virtual database via the General Packed Radio Service (GPRS) communication device as it was the easiest method to continuously observe the data flow by means of an internet connection without frequent visits to the equipment.

The real-time data have been collected from three different critical components to compare the overall performance of the autoclave. Considering that two components among the three present similar physical performance parameters with the same indicators even though they serve different activities, during our experiment we have considered data from only two components. The four variables’ dataset from which a short sample is shown in Table 1 consists of Temperature (Temp.), Vibration (Vibr.), and two Current (Curr.) flows from the different sources (A and B), which is considered to build our models.

Since the equipment does not operate all the time, the data were only gathered during operation times.

### 2.2. Data Preprocessing and Labeling Using Fuzzy Expert System

For the PdM precision and decision making on maintenance activities, the supervised predictive analytics tools are quite helpful. This requires the preprocessed data [25] such as cleaned, prepared, and labeled data.

#### 2.2.1. Data Preprocessing

Since for real-time application at the edge, the raw data from the sensors are fed immediately to the predictive analytics model on the device for processing and analytics, every step performed in the data preprocessing phase has to be captured and coded onto the microcontroller to be executed before data are able to be supplied to the TinyModel. As a result, it is necessary to maintain the originality of the data. As a result, the data processing may not require more cleanup. Only missing data and data normalization are taken into consideration.

#### 2.2.2. Recall of Fuzzy Expert System in Predictive Maintenance

Considering that all decisions on maintenance activities are taken via the maintainer’s expertise, there is no a single way to assume the health status of the equipment without assessing each part of it, and that the shortage from any component may affect the overall performance of the equipment or cause a failure. As a result, we needed to use a tool that may put together different health statuses from different critical components of a single piece of equipment to determine the maintenance priorities.

Fuzzy logic, an artificial intelligence software proposed by Zadeh (1965) [26], is adopted as a tool to facilitate the transformation of the maintainers’ expertise into an automated expert system that will provide a continuous observation of the equipment state and accordingly plan and prioritize maintenance activities before any unplanned downtime can occur.

For complex industrial equipment with many components, including uncertain and imprecise behavior, there is a risk that unplanned downtime may occur for the whole system. To address this problem, fuzzy logic is a powerful tool for modeling and controlling this type of equipment [27]. Referring to the literature [28,29,30,31,32,33], fuzzy logic has been used in maintenance as a decision-making, scheduling, and hazard-level reckoning tool.

Based on the maintainers’ expertise as well as long-time continuous monitoring of the equipment and their components’ health status vis a vis their physical conditions and associated performance, we labeled our conditional data from sensors using the Fuzzy Expert System, which is simple to learn and use, as it does not require a speculative model.

**Figure 3:**
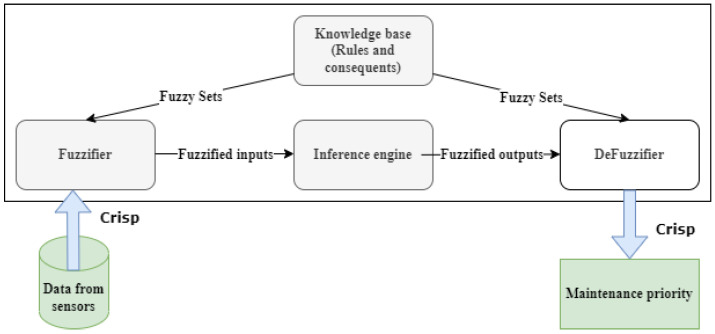
Fuzzy inference system.

The diagram of the Fuzzy Expert System [34] in Figure 3 shows that the data from the sensors are fed to the fuzzifier to be converted into fuzzy input sets with some extent of membership varying in the interval of [0, 1]. Each value in the [0, 1] interval denotes the degree of ambiguity in the set—0 means that the value does not fit the fuzzy set; 1 represents a peak value in the set. The fuzzified inputs set value (*F*(*i*)) is computed using a sigmoid function in Equation (1). The function presents the symmetry property described in Equations (2) and (3).

(1) $$F\left(i\right)=\frac{1}{1+e^{-}1}$$

(2) F(i)+F(−i)=1

(3) $$\left(F\left(i_{1}\right)+F\left(-i_{1}\right)\right)\times \left(F\left(i_{2}\right)+F\left(-i_{2}\right)\right)\times \ldots \times \left(F\left(i_{n}\right)+F\left(-i_{n}\right)\right)=1$$

The fuzzified input set is directed to the Mamdan inference engine [35] to be weighed by mapping the defined fuzzy sets, which are well-matched to experienced human operators that map precedent rules (created using IF, both AND and OR operators, THEN syntax) and consequences in a knowledge base. The fuzzy set (*X*) is presented as a group of tidy pairs, as shown in Equation (4) and the mapping function (*M*(*i*)) in Equation (5). The inference aggregates the output (crisp value), which is transformed into a real-world output by the defuziffier.

(4) X=(i,M(i)),i⊂I

(5) M(i)=I→[0,1]

where *X* is a fuzzy set, *M*(*i*): Membership function, *i*: element belongs in the universe of discourse and *I*: universe of discourse.

#### 2.2.3. Data Labeling (RUL) Using a Fuzzy Expert System

Considering that preventive maintenance is regularly conducted to keep up the equipment, reaching the downtime state of the equipment is not feasible; thus, the remaining useful time is defined as the remaining time to reach the very-high maintenance priority zone and the remaining time is calculated in terms of days. The possible scenarios from input variable membership to create fuzzy rules are assessed in order to build a fuzzy system that can figure out the maintenance activities priority liable to the health status of different components of the equipment. The maintenance priorities are then used to compute the remaining useful life (RUL) of the entire equipment. The arithmetical functions in Python are used to determine the RUL at each row of data by considering a current data point to compute the remaining time until the data point, which belongs to the very-high maintenance priority zone.

**Figure 4:**
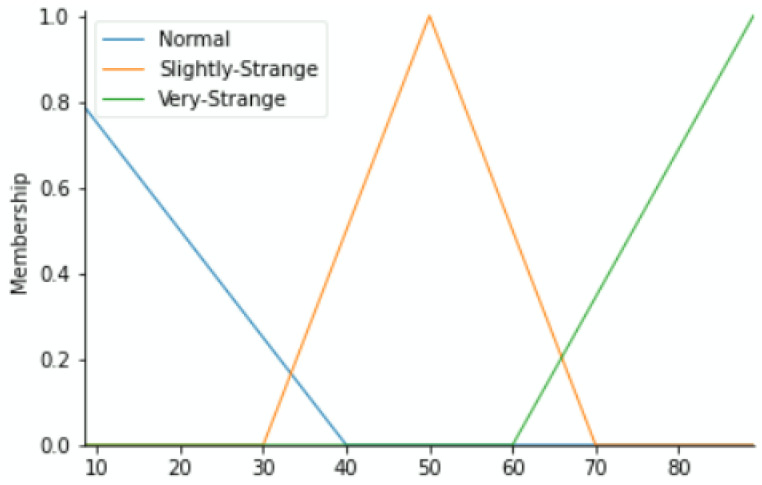
Temperature variable.

**Figure 5:**
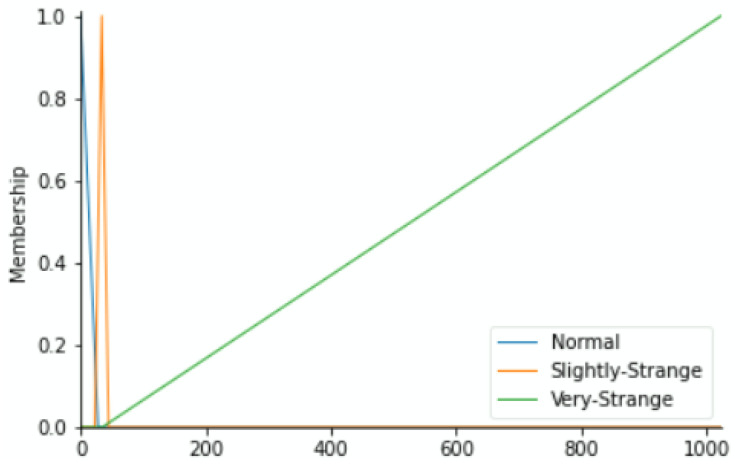
Vibration variable.

**Figure 6:**
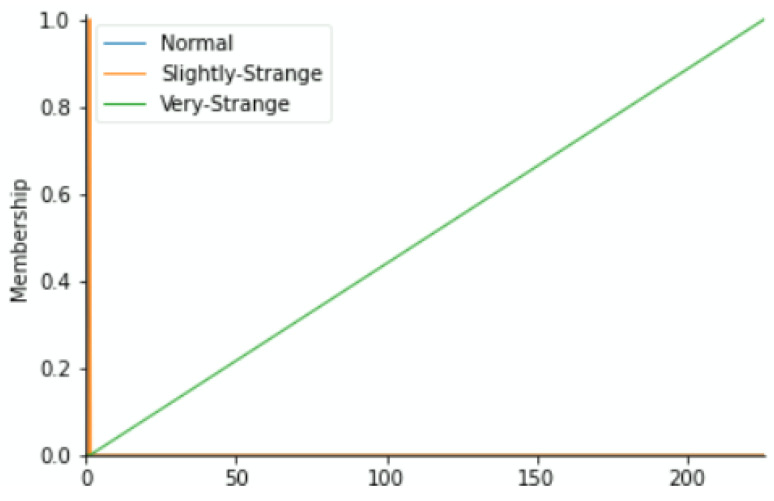
Current (A) variable.

**Figure 7:**
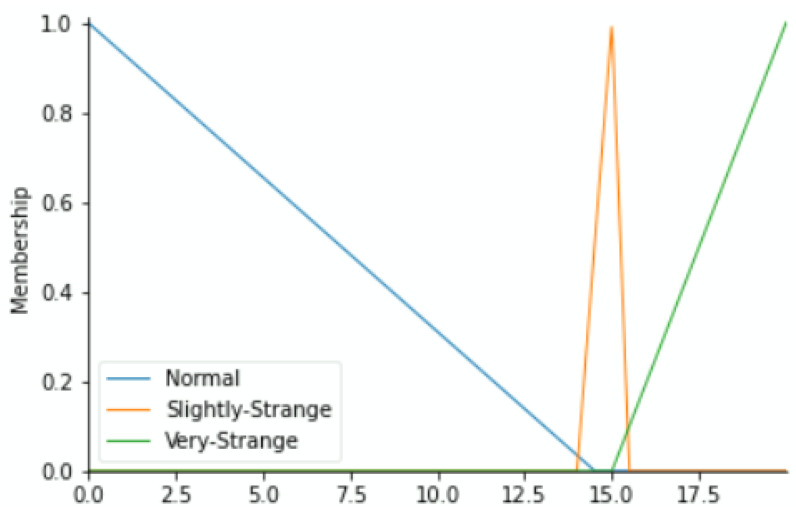
Current (B) variable.

**Figure 8:**
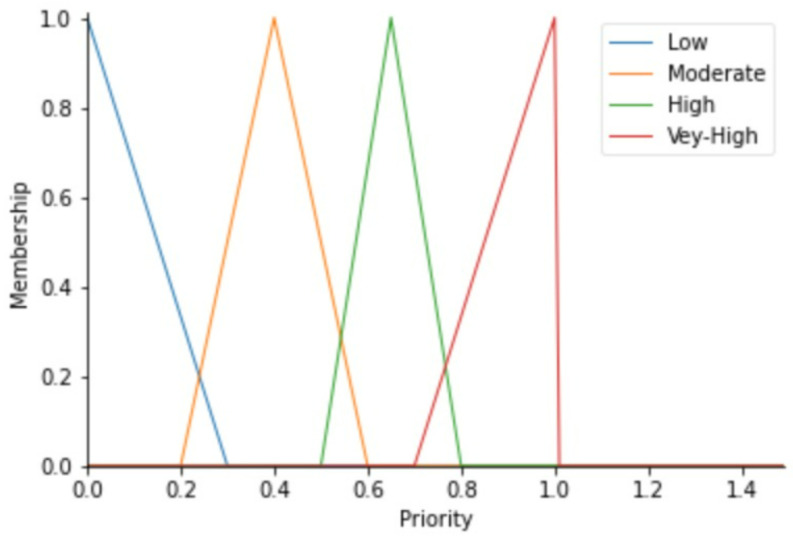
Maintenance priority variables.

To label our data, we reflected on the fact that each component may push the equipment to downtime. Based on the historical methods used to detect errors during regular preventive maintenance and expert inspections as well as their results, we have classified the health status into the Fuzzy Expert System’s linguistic variables of each component versus the value range of its working condition parameters as Normal, Slightly Strange, and Very Strange. The output variable (maintenance action priority) zones shall depend on the fuzzy membership functions of status from the four variables. By means of the triangular membership function, Figure 4, Figure 5, Figure 6 and Figure 7 show the linguistic variable as the health status ranges (on the *X*-axes) of different parameters—Figure 8 summarizes the maintenance priority output. The *X*-axes and *Y*-axes present the ranges of maintenance priority and the degree of priority, respectively.

The maintenance priority linguistic variables recall the urgency levels as either low, moderate, high, or very high, which designate the need for maintenance action as either no action, far future, near future, or immediate maintenance action, respectively.

From the maintenance priority calculated by the Fuzzy Expert System, we labeled our dataset by computing the remaining useful life (RUL). The RUL is calculated using functions in Python that consider the current state and compute the probable time to attain the neighbor data point belonging in the risky zone, which is indicated by very-high priority. The RUL is calculated in terms of the number of days remaining for the equipment to operate continuously before entering the risky zone. Adding to the raw data in Table 1, Table 2 shows randomly picked rows from the dataset with two added columns consisting of maintenance priority (M. Priority) generated through fuzzy and different values of calculated RUL as a label of our dataset. The priority varies in a normal fuzzy output range of [0, 1], whereas the RUL is calculated in terms of days and the maximum is 22 without any maintenance.

## 3. Predictive Analytics Models (LSTM and Model from Edge Impulse)

Replying to the shortage in continuous and real-time availability of industrial equipment health states with the purpose of providing predictive maintenance solutions at the edge in real-time, and by taking consideration of the LSTM model prediction performance on real or sequential data from the literature and the currently booming Edge Impulse platform that provides a single way to develop and deploy real solutions on the edge device, both LSTM and the model from Edge Impulse are assessed in this work.

### 3.1. Long Short-Term Memory (LSTM)

In the TensorFlow backend, using Python, data were preprocessed, labeled using fuzzy logic, as described in Section 2.2.2, and later through the Keras library [23], which was used to build the sequential long short-term memory (LSTM) model.

The long short-term memory (LSTM) is an improved cell of recurrent neural networks (RNN) [36], which was developed to mitigate the gradient vanishing presented by the vanilla RNN. RNN, in its structure, presents a short memory to keep a current iteration output. It then feeds this output as an input to the following iteration. This makes RNN suitable to process the time series data by keeping a memory of the last iterations and recognizing the dependencies between iterations.

**Figure 9:**
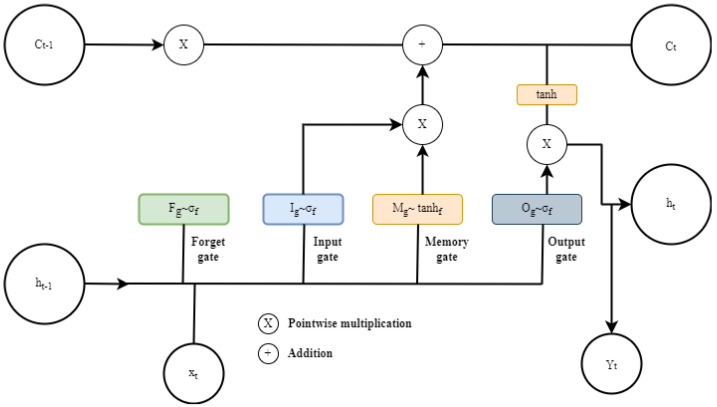
LSTM cell structure.

Bearing to the ordinary short-term memory of the RNN, LSTM adds long-term storage capability and gates that allow the algorithm to reflect on the long-term dependencies from the data of the past iterations and also increase the learning process. This makes LSTM more suitable to handle sequential problems, especially with time series data. The information to be kept or forgotten from the LSTM memory is determined by its gates. The LSTM cell structure is shown in Figure 9, which presents four collaborating gates, namely input (I), forget (F), memory cell (M), and output (O) gates, which replace the hidden neurons of the ordinary RNN.

The four gates also represent the four layers through the sigmoid (σ) and tangent activation (tanh) functions that compute each gate’s task in order to update the cell state memory (*C_t_*) and to control the output as well.

Based on the previously hidden state information *hx* − 1 and current iteration *X_t_* at time *t*, using the sigmoid function, the forget gate compute Equation (6), to decide on which information in the memory state to keep or to throw away.

(6) $$\begin{array}{c} f_{t}=\sigma \left(W_{f}x_{t}+W_{h}h_{t}-1+b_{f}\right) \end{array}$$

The sigmoid layer output varies between 0 and 1 and if the output is closer or equal to zero, all information is thrown out. After the forget layer decides, the input gate decides on the new candidate data for the next layer. It performs Equation (7) using the sigmoid function to quantify the new information from the new iteration to update the memory state (*M_t_*).

(7) $$\begin{array}{c} i_{t}=\sigma \left(W_{i}x_{t}+W_{h}h_{t}-1+b_{i}\right) \end{array}$$

On the other side, the tanh layer residing in the memory gate generates a vector of new memory value (*M_t_*) over Equation (8). The two gates’ outputs are then combined and pointwise multiplied in Equation (9) to create an update to the cell memory state (*C_t_*).

(8) $$\begin{array}{c} m_{t}=\phi \left(W_{m}x_{t}+W_{m}h_{t}-1+b_{m}\right) \end{array}$$

(9) $$\begin{array}{c} C_{t}=f_{t}\times C_{t-1}+i\times m_{t} \end{array}$$

The output gate applies the tanh function to the cell memory, then computes Equation (10) to decide on the information to be output. Equation (11) is then computed to determine the hidden information for the next iteration.

(10) $$\begin{array}{c} o_{t}=\sigma \left(W_{o}x_{t}+W_{o}h_{t}-1+b_{0}\right) \end{array}$$

(11) $$\begin{array}{c} h_{t}=o_{t}\times \phi \left(c_{t}-1\right) \end{array}$$

After all computation into the cell, it produces the predicted information (*Y_t_*) by performing Equation (12):

(12) $$\begin{array}{c} Y_{t}=w_{0}h_{t}+b_{0} \end{array}$$

Notation: *F*: forget, *I*: input, *M*: memory cell, *O*: output gate, *t*: time at current iteration, *t* − 1: time at previous iteration, σ: Sigmoid activation function, *W*: connection weight matrix allied to the gate hidden state, *x_t_*: current input at time *t*, *h_t_* − 1: hidden state from the previous iteration, *b*: bias, and *C_t_* cell memory state at time *t*.

### 3.2. Predictive Analytics Model from Edge Impulse

Edge Impulse is the novel platform in the era of machine learning with the purpose of providing embedded machine learning solutions on edge applications [22]. Adding to other machine learning development platforms, Edge Impulse provides the simplest way to collect data using either built-in or outer sensors in smart devices, such as mobile and embedded devices. Edge Impulse also helps in analyzing the data, designing and testing the model, as well as providing the deployable version of the model without much experience in coding. It also allows customized data and the ability to customize the model design.

## 4. Results and Discussion

The intention of this section is to build and compare the two competent sequential models that drive us to adopt a suitable predictive analytical model for IoT-based predictive maintenance real-time application. From the literature, LSTM was mainly used and performed well on time series data. In contrast, the Edge Impulse platform has provided a simplified way to build and deploy a new model on edge applications. Consequently, both the LSTM and model from Edge Impulse were designed, trained, and evaluated to learn real-time data collected from autoclave equipment for our experiment. Both models were then converted into TinyModel versions and deployed on the edge through simulation using unseen real-time data.

### 4.1. LSTM Model Structure and Performance Metrics

Using the Keras deep learning library [23], which runs on the TensorFlow platform [37] in Python, we built the LSTM analytics predictive model. Our dataset contains a total of 126,333 data points taken with an equal interval of one minute between two data points. As LSTM refers to the previous data to figure out the convenient function of new data, we set data to be sampled into a small sample of 60 data points, whereby LSTM shall learn before concluding on the next prediction point. The sample size was fixed based on the length of time used by the equipment to complete a single operation cycle and that the health status assessment could be summarized at least after each operation cycle.

**Figure 10:**
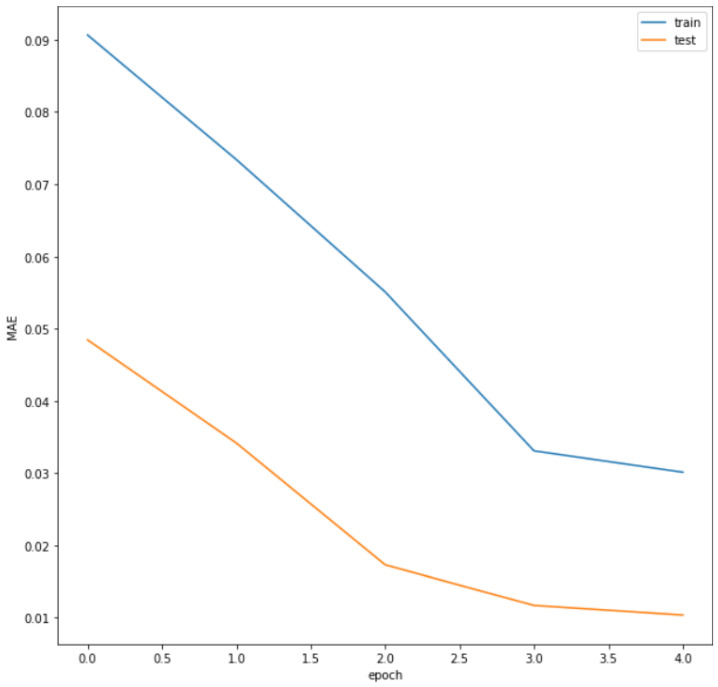
LSTM model loss.

To fit the LSTM structure, our data were arranged into a three-dimensional array format and split into two parts, the train and test datasets, with portions of 80% and 20%, respectively. The data format loaded into the LSTM to train and after to test is shown in Figure 10, where the priority and RUL (two last columns, respectively) were used to build a predictive analytics model.

Since each model performs according to the applied hyperparameters, we iterate the training and testing phases using different parameters to obtain the best results evaluated by assessing loss and model performance accuracy through mean squared error (MSE) and coefficient of determination (R) metrics for both training and testing datasets.

The adopted model structure, hyperparameters, and performance result metrics’ values at a minimum loss are presented in Table 3.

Our model fits the regression line at 77%, which is reasonably good since the data may vary from normal to worse values; we note that the far data from the regression line could not be considered as an outlier if it belongs to the equipment condition parameters’ range. The total train loss of 0.029 and total test loss of 0.01 are small, which is good for the model’s performance.

Figure 10 presents the model loss trend. To determine the best model performance values for minimum loss and overfitting, at each hyperparameter value, the model was tested on both datasets. It is seen that the losses for the test dataset are lower than that of train dataset and overfitting was not present. In addition, both model training and testing losses are lower as the epochs increase and keep steady at closer points.

**Figure 11:**
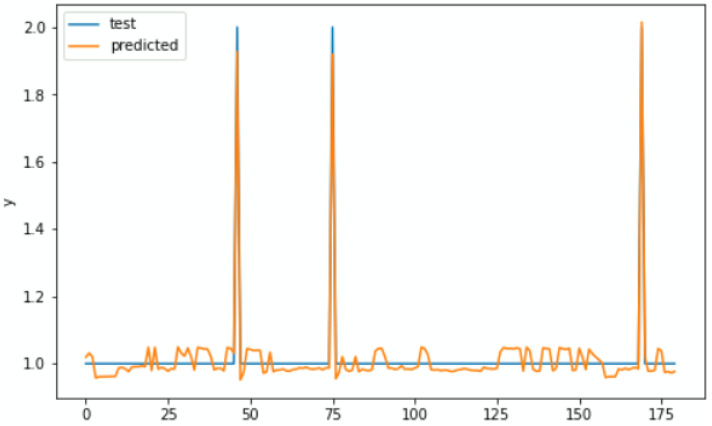
Actual versus predicted RUL.

To evaluate the real and predicted RUL relationship, the test dataset was used. Figure 11 shows that both real and predicted RUL (on *Y*-axes) are reasonably closer when you keep rounding a float number to an integer. The RUL in our real data is rounded to the closer integer at each time point whereas the predicted results are kept in floats.

#### Model from Edge Impulse Structure and Performance Metrics

The Edge Impulse platform provides a well-structured and simple step to build a model. It allows users to upload different types of preprocessed data.

**Figure 12:**
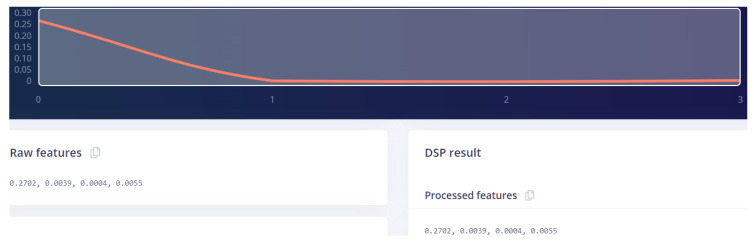
Data presentation in Edge Impulse.

For this experiment on a predictive model, the raw data were uploaded and indicated as time series data. Since our data were used for regression problems, the data upload technique may differ from the familiar classification method. The dataset structure requires separate files, each named under its label for all data points. Figure 12 presents a single sampled data point and its corresponding features as each data point of our dataset is made by four variables and named on its specific label.

Forwarding to the model building, we start with the default settings and built-in neural network (NN) architecture in Edge Impulse, and we keep tuning the settings and retraining to obtain a best model that may fit our data with the least amount of loss. Table 4 illustrates the adopted optimal prior model parameter settings and the architecture of the NN block.

The hidden dense layers are fully connected. Within the current version of the developed Edge Impulse platform for regression problems, the model output always appears in classes. With the optimum specifications in Table 4, using the validation dataset, the achieved minimum loss reaches 0.11.

**Figure 13:**
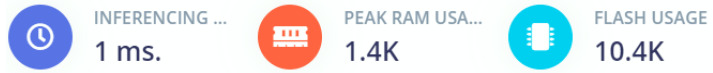
On Device Performance.

As the Edge Impulse is specifically designed to build models for real-time application on the edge, after each training and validation set, the built model summarizes the model performance on the device at the edge, as shown in Figure 13.

**Figure 14:**
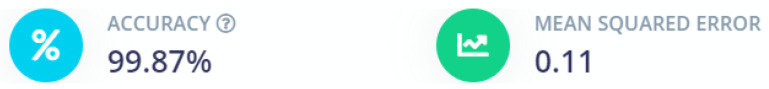
Edge Impulse model testing results.

Figure 14 shows the specification of the device at the edge to host the TinyModel. The model testing results show a good model performance of 99.87% and MSE of 0.11.

**Figure 15:**
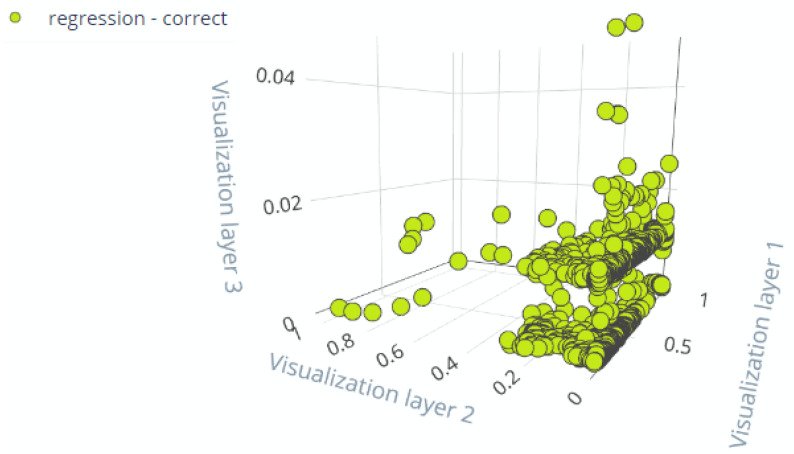
Evaluation data presentation.

The results in Figure 15 show that most of the performance data from our equipment fall into class one of the RUL, which is similar to actual RUL from raw data.

### 4.2. TinyModel

For both the LSTM model and model from Edge Impulse, the TinyModel is obtained by converting the ordinal neural-network-based model to TinyModel. From Edge Impulse, the conversion process is integrated into the platform. In contrast, for the LSTM model, we used the TensorFlow-Lite (TF-Lite) converter to convert the ordinary Keras LSTM to TF-Lite TinyLSTM.

Both conversions methods require specifying the type of device that will host the TinyModel. Considering the industrial constraint of energy consumption, we chose the Arduino Nano BLE Sense [38], which was purposely designed to have power saving features for IoT-based edge applications.

To learn more about the needed embedded device to host the model into Edge Impulse, we optimize and compile the model for deployment to check the final recommended specification of targeted embedded devices. Since the TF-Lite does not provide the summary of the needed device specifications additional tools will be required to assume the needed deployment memory and possible latency.

**Figure 16:**
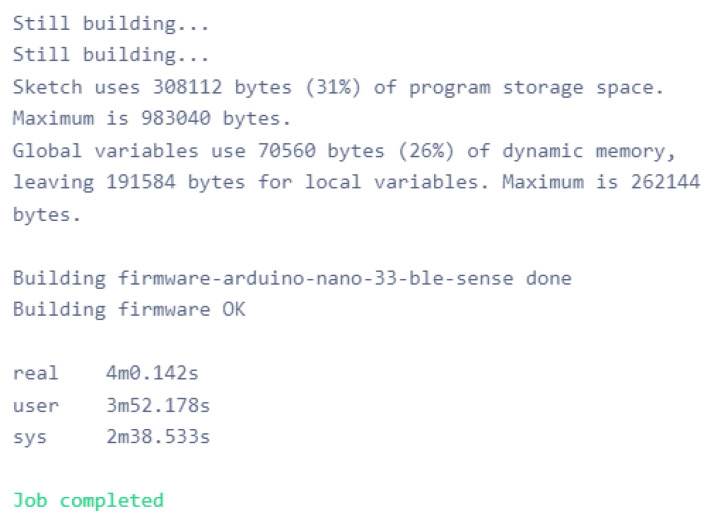
TinyModel firmware for Arduino Nano BLE Sense.

Finally, the TinyModels for both LSTM and the model from Edge Impulse were built, and the source files were downloaded to be installed and deployed on the embedded device. Figure 16 shows the TinyModel firmware for Arduino Nano BLE Sense built from Edge Impulse.

### 4.3. Simulating the Deployment and Inference Creation

**Figure 17:**
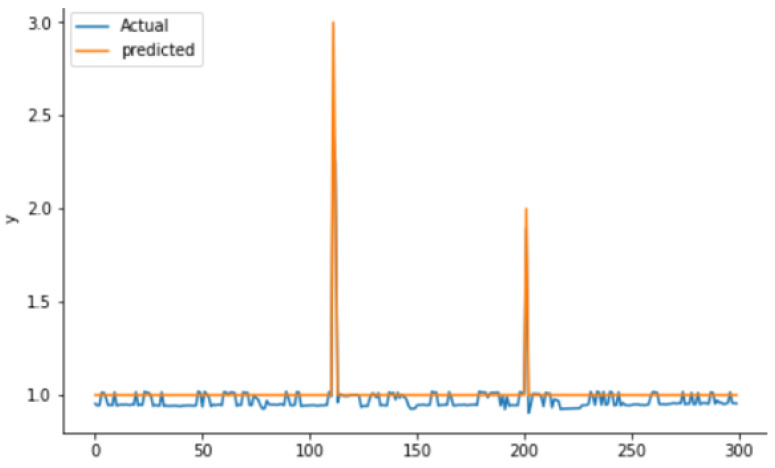
Deployment simulation results for TinyLSTM-Model.

**Figure 18:**
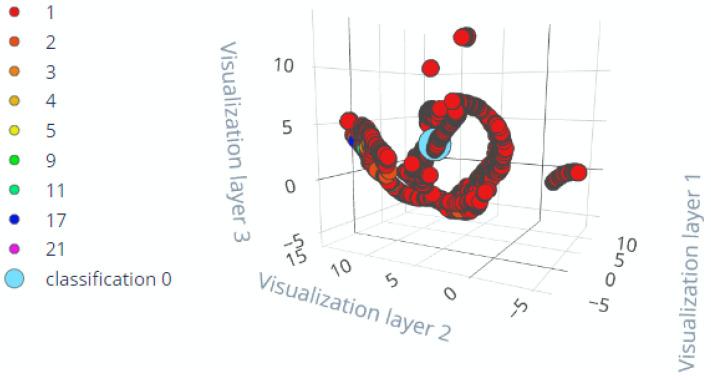
Deployment simulation results for TinyEI-Model.

Both TinyModel’s (TinyLSTM & TinyModel_EI) deployment were simulated using unseen data gathered from the same equipment. Prior to feeding data to the simulator, all preprocessing activities performed on training and testing data were considered and must be coded onto the microcontroller to be processed before reaching the TinyModel. The obtained simulation results from both TinyModels are shown in Figure 17 and Figure 18, respectively, for TinyLSTM and TinyModel_EI.

Deployment simulation results were shown to have almost the same model performance as the results obtained when evaluating the models. To evaluate the similarities between actual and predicted outputs, we compared actual and predicted RUL and found no differences. To facilitate the maintainers that observe the detailed condition status of their equipment, we must determine the critical status of the overall equipment. Adding to Figure 17 and Figure 18, Table 5 shows the real physical conditional values from the different components of the equipment and predicted RUL in order to determine the overall equipment status. The values from components that may cause the downtime and RUL in the last column are highlighted in red.

Depending on the maintainers’ demand, physical conditional values in the same health status could be given the same color code to help them observe and fully explain the reason behind the predicted RUL.

### 4.4. Models’ Comparison

The comparison consists of three main components, which are the coding platform and data processing in Table 6, model structure and performance metrics in Table 7, and TinyModel conversion and deployment in Table 8.

Summarizing the comparisons in Table 6, Table 7 and Table 8, both models performed well on data with minimum losses and a slight difference in losses from one model to another. In contrast, through comparing the models building up to TinyModel processes, LSTM requires significant experience in coding and requires much more training time than the Edge Impulse model. Edge Impulse limits the user to customizing their own detailed graphical presentations, but it is much more user friendly and easier for people with limited programming skills. Coming to the deployment on edge, Edge Impulse provides some estimated information, such as required device memory and processing latency, which is not given by the TensorFlow Lite platform. From the deployment simulation, referring to latency, LSTM may also reflect the higher power consumption than TinyModel_EI. Hence, TinyModel_EI is much easier to develop and a more suitable real-time model for deployment than TinyLSTM.

## 5. Conclusions

The RNN models, specifically its LSTM, have been appreciated in the literature based on their ability to perform well on sequential problems. Looking to the RUL in the era of IoT-based predictive maintenance on the edge, LSTM development until its TinyModel deployment on the edge is compared to the new vibrant model from Edge Impulse designed to support the machine learning model on the edge. Both models are designed, trained, and tested using the real-time data collected from industrial complex equipment with the purpose to assess and adopt a suitable and easiest model for real-time predictive maintenance applications on edge.

Considering the impact of each component of the equipment on the overall health of complex equipment, the Fuzzy Logic Expert System, which is based on human expertise, is utilized to mark the maintenance priority level of the equipment by combining different states from its different components and predefined rules to detect its different health levels. The fuzzy output is used to compute the actual RUL for model training and testing.

The evaluation and deployment simulation results from both models proved the good performance with slight regression loss of 0.01 and 0.11, respectively, for the LSTM and the model from Edge Impulse. Since both models perform well on real-time data, the adoption of the best model is based on the overall process from model build-up, up to its deployment on the edge and information on TinyModel deployment. The deployment on the edge is much easier for the Edge Impulse platform compared to LSTM, as LSTM is built using TensorFlow, requires an experienced developer, takes a significant amount of time to develop, and requires TensorFlow Lite for the conversion to a TinyModel version; therefore, we recommend the adoption of TinyModel from Edge Impulse in the era of automated and continuous real-time predictive maintenance.

To extend this work, the various conditional parameters from different components of the equipment shall be observed to have fully continuous monitoring of the equipment RUL.

## Figures and Tables

**Table 1:** Dataset sample presentation.

Temp. (°C )	Vib. (mm/s)	Curr. A (mA)	Curr. B (mA)
33.44	48	0.12	0.12
33.88	46	0.08	17.53
30.56	16	2.72	0.10
32.50	11	0.03	0.12
35.56	7	0.01	0.12
35.88	47	0.01	0.10
43.63	47	0.06	0.12

**Table 2:** Labeled dataset.

Temp. (°C )	Vib. (mm/s)	Cur. A (mA)	Cur. B (mA)	M. Priority (0 to 1)	RUL (Days)
89	12	2.53	17.65	0.90	1
47	6	6.07	0.01	0.86	2
34.81	12	4.89	0.12	0.86	3
44.88	6	2.79	0.03	0.86	4
31.44	9	5.21	0.15	0.86	5
33.75	0	3.56	0.11	0.86	6
26.87	0	0.1	15.45	0.89	7
28.25	50	0.13	0.05	0.86	8
38	7	0.13	0.12	0.38	9
34	6	0.11	0.11	0.31	10
37.88	8	0.1	0.12	0.38	11
30.31	0	0.1	0.12	0.15	12
30.31	10	0.1	0.12	0.15	13
29.25	0	0.08	0.12	0.13	14
29.25	0	0.08	0.11	0.13	15
29.25	0	0.13	0.1	0.13	16
28.44	0	0.1	0.11	0.13	17
25	0	0.13	0.33	0.12	18
28.5	0	0.03	0.32	0.13	19
28.5	0	0.13	0.11	0.13	20
30.19	10	0.1	0.11	0.15	21
30.18	9	0.11	18.2	0.89	22

**Table 3:** Model structure metrics and performance values.

Parameters	Optimum Metrics’ Value
Model training dataset portion	80%
Model evaluation dataset portion	20%
Model Type	Sequential
LSTM layer	32 neurons
Hidden Dense layer	16 neurons
Dropout packaging	0.2
Output layer (Dense)	1 neuron
Optimizer	Adam
Learning rate	0.001
Epoch	5
Performance metrics	MSE (Mean Square Error) and Coefficient of determination $R^{2}$
Batch size	16
Time step window	60
Train MSE	0.0295
Test MSE	0.01
$R^{2}$	0.77

**Table 4:** Model parameter settings and neural network block architecture.

Parameters	Specifications
Training Cycles	10 Cycles
Training dataset	80% of the entire dataset
Testing dataset	20% of the entire dataset
Validation dataset (to be used during training)	20% of the entire dataset
Learning rate	0.005
Activation	ReLu
Batch size	32
Epoch	10
Loss function	Mean Squared Error (MSE)
Model type	Sequential
Input layer	4 features
Hidden Dense layer at first level	20 neurons
Hidden Dense level at second level	10 neurons
Output layer	1 class (1 neuron—no Activation)

**Table 5:** Real-time data and predicted RUL.

Temp. (°C )	Vib. (mm/s)	Cur. A (mA)	Cur. B (mA)	Actual RUL (Days)
50.56	45	0.14	17.97	1
54.31	25	2.63	0.01	1
54.31	55	2.65	0	1
55.13	127	2.71	18.07	1
47.69	50	0.14	18.09	1
41.44	42	0.14	18.23	1
37.88	48	0.14	18.12	1
36	70	0.14	18.06	1

**Table 6:** Coding platform and data processing for LSTM and Model from EI.

Element	For LSTM Model	For Model from Edge Impulse
Model building platform	TensorFlow	Edge Impulse
Free version of platform	No limitation on data size and training time but keep confirming the work in progress	Limited data size and training time
Library	Keras [23]	Keras [23]
Data preprocessing	In same platform	Out of Edge Impulse

**Table 7:** Model structure and performance metrics.

Element	LSTM Model	Model from Edge Impulse
Model Type	Sequential	Sequential
Model structure	Based on Neural networks block	Based on Neural networks block.
Model build up	Customized by a developer	There is a proposal of standardized inbuilt model which could be customized.
Training time for same dataset	Long	Short
Regression performance metrics	To be defined by the developer	Defaulted as MSE and can be customized in Expert mode.
Outputs representation	Customized by the developer depending on the metrics to be presented	Defaulted and limited
Activation	To be defined and mostly ReLu for regression model (Keras standardized)	Defaulted as ReLu and can be customized in Expert mode
Ordinary model building simplicity	Depends on the experience of the developer	Standardized inbuilt model may perform well on the data and in case of improvement, it is easy even for less experienced developer
Regression output	Single Value	Class
Overfitting possibility	Much	Less
Model Train loss (MSE)	0.0295	0.11 on validation dataset
Model Test loss (MSE)	0.0092	0.11
Model performance	$R^{2}$: 77%	Accuracy: 99.87%

**Table 8:** TinyModel conversion and deployment.

Element	LSTM Model	Model from Edge Impulse
Converting the ordinary model to TinyModel	Using TensorFlow Lite	Inbuilt conversion
TinyML device required memory	Not assumed	Both RAM and ROM (flash) memory are estimated for a given edge device.
Latency of the TinyModel on IoT device	Not assumed	Estimated by Edge Impulse platform. Latency equals to 1 ms in our case Cortex-M4F—64 MHz)
Microcontroller for edge deployment	On Choice: in this case Arduino Nano BLE Sense is chosen	On Choice: in this case Arduino Nano BLE Sense is chosen

## Data Availability

The data used to support the findings of this work are available from the fist author upon request.

## Abbreviations

The following abbreviations are used in this manuscript:

AI	Artificial Intelligence
EI	Edge Impulse
IoT	Internet of Things
LSTM	Long Short-Term Memory
MSE	Mean Squared Error
PdM	Predictive Maintenance
RNN	Recurrent Neural Networks
RUL	Remaining Useful Life
TF	TensorFlow
TinyML	Tiny Machine Learning

## References

[1] Ran Y., Zhou X., Lin P., Wen Y., Deng R. (2019). A Survey of Predictive Maintenance: Systems, Purposes and Approaches. arXiv.

[2] Lee J., Wu F., Zhao W., Ghaffari M., Liao L., Siegel D. (2014). Prognostics and health management design for rotary machinery systems—Reviews, methodology and applications. Mech. Syst. Signal Process..

[3] Aydemir G., Acar B. (2020). Anomaly monitoring improves remaining useful life estimation of industrial machinery. J. Manuf. Syst..

[4] Xia T., Song Y., Zheng Y., Pan E., Xi L. (2020). An ensemble framework based on convolutional bi-directional LSTM with multiple time windows for remaining useful life estimation. Comput. Ind..

[5] Wang B., Lei Y., Li N., Yan T. (2019). Deep separable convolutional network for remaining useful life prediction of machinery. Mech. Syst. Signal Process..

[6] Wu Y., Yuan M., Dong S., Lin L., Liu Y. (2018). Remaining useful life estimation of engineered systems using vanilla LSTM neural networks. Neurocomputing.

[7] Li H., Zhao W., Zhang Y., Zio E. (2020). Remaining useful life prediction using multi-scale deep convolutional neural network. Appl. Soft Comput. J..

[8] Sikorska J.Z., Hodkiewicz M., Ma L. (2011). Prognostic modelling options for remaining useful life estimation by industry. Mech. Syst. Signal Process..

[9] Ayvaz S., Alpay K. (2021). Predictive maintenance system for production lines in manufacturing: A machine learning approach using IoT data in real-time. Expert Syst. Appl..

[10] Alipour M., Mohammadi-Ivatloo B., Zare K. (2015). Stochastic Scheduling of Renewable and CHP-Based Microgrids. IEEE Trans. Ind. Inform..

[11] Li X., Ding Q., Sun J.Q. (2018). Remaining useful life estimation in prognostics using deep convolution neural networks. Reliab. Eng. Syst. Saf..

[12] Benkedjouh T., Medjaher K., Zerhouni N., Rechak S. (2013). Remaining useful life estimation based on nonlinear feature reduction and support vector regression. Eng. Appl. Artif. Intell..

[13] Celikmih K., Inan O., Uguz H. (2020). Failure prediction of aircraft equipment using machine learning with a hybrid data preparation method. Sci. Program..

[14] Zhang X.H., Kang J.S. Hidden Markov models in bearing fault diagnosis and prognosis. Proceedings of the 2010 Second International Conference on Computational Intelligence and Natural Computing.

[15] Kotsiopoulos T., Sarigiannidis P., Ioannidis D., Tzovaras D. (2021). Machine Learning and Deep Learning in smart manufacturing: The Smart Grid paradigm. Comput. Sci. Rev..

[16] Thanasis J., Ma Y., Zhang L., Gao R.X., Wu D. (2018). Deep learning for smart manufacturing: Methods and applications. J. Manuf. Syst..

[17] Zhou Y., Hefenbrock M., Huang Y., Riedel T., Beigl M. (2021). Automatic Remaining Useful Life Estimation Framework with Embedded Convolutional LSTM as the Backbone. Lect. Notes Comput. Sci..

[18] Li J., Li X., He D. (2019). A Directed Acyclic Graph Network Combined With CNN and LSTM for Remaining Useful Life Prediction. IEEE Access.

[19] Elsheikh A., Yacout S., Ouali M.S. (2019). Bidirectional handshaking LSTM for remaining useful life prediction. Neurocomputing.

[20] Yu Y., Hu C., Si X., Zheng J., Zhang J. (2020). Averaged Bi-LSTM networks for RUL prognostics with non-life-cycle labeled dataset. Neurocomputing.

[21] Compare M., Baraldi P., Zio E.T. (2020). Challenges to IoT-Enabled Predictive Maintenance for Industry 4.0. IEEE Internet Things J..

[22] Advanced ML for Every Solution. https://www.edgeimpulse.com.

[23] About Keras. https://keras.io/about/.

[24] Niyonambaza I., Zennaro M., Uwitonze A. (2020). Predictive maintenance (Pdm) structure using internet of things (iot) for mechanical equipment used into hospitals in Rwanda. Futur. Internet.

[25] Bekar E.T., Nyqvist P., Skoogh A. (2020). An intelligent approach for data pre-processing and analysis in predictive maintenance with an industrial case study. Adv. Mech. Eng..

[26] Zadeh L.A. (1965). Fuzzy sets. Inf. Control.

[27] Mihigo I.N., Zennaro M., Uwitonze A. Enhancing the Priority for the Maintenance Activities of the Hospitals’ Mechanical Equipment Using the Fuzzy Expert System. Proceedings of the 13th EAI International Conference, AFRICOMM 2021.

[28] Baban M., Baban C.F., Moisi B. A Fuzzy Logic-Based Approach for Predictive Maintenance of Grinding Wheels of Automated Grinding Lines. Proceedings of the 23rd International Conference on Methods and Models in Automation and Robotics MMAR.

[29] Baban M., Baban C.F., Suteu M.D. (2019). Maintenance Decision-Making Support for Textile Machines: A Knowledge-Based Approach Using Fuzzy Logic and Vibration Monitoring. IEEE Access.

[30] Kumar E.V., Chaturvedi S.K. (2011). Prioritization of maintenance tasks on industrial equipment for reliability: A fuzzy approach. Int. J. Qual. Reliab. Manag..

[31] Borjalilu N., Ghambari M. (2018). Optimal maintenance strategy selection based on a fuzzy analytical network process: A case study on a 5-MW powerhouse. Int. J. Eng. Bus. Manag..

[32] Andrew A., Kumanan S. (2020). Development of an intelligent decision making tool for maintenance planning using fuzzy logic and dynamic scheduling. Int. J. Inf. Technol..

[33] Gallab M., Bouloiz H., Alaoui Y.L., Tkiouat M. (2019). Risk Assessment of Maintenance activities using Fuzzy Logic. Procedia Comput. Sci..

[34] Jang J.R. (1993). ANFIS: Adaptive Network-Based Fuzzy Inference System. IEEE Trans. Syst. Man Cybern..

[35] Fuzzy Logic—Controls, Concepts, Theories and Application: A Mamdani Type Fuzzy Logic Controller. www.intechopen.com.

[36] Hochreiter S., Schmidhuber J. (1997). Long Short-Term Memory. Neural Comput..

[37] TensoFlow Home Page. https://www.tensorflow.org/.

[38] Arduino Nano Sense Ble. https://docs.arduino.cc/hardware/nano-33-ble-sense/.

